# Neurotrophic factors in multiple sclerosis

**DOI:** 10.3389/fimmu.2025.1654603

**Published:** 2025-08-27

**Authors:** Fabian Güner, Veit Rothhammer

**Affiliations:** ^1^ Department of Neurology, University Hospital Erlangen, Friedrich-Alexander University Erlangen-Nürnberg, Erlangen, Germany; ^2^ Deutsches Zentrum Immuntherapie (DZI), University Hospital Erlangen, Erlangen, Germany

**Keywords:** astrocytes, protective, neurotrophic factor, neuroinflammation, multiple sclerosis, BDNF, NGF, HB-EGF

## Abstract

Smoldering inflammation and neurodegeneration, primarily driven by intraparenchymal immune cell activation and glial dysfunction, remains a major therapeutic challenge in Multiple Sclerosis (MS) and contributes largely to disability progression. Current disease-modifying therapies effectively decrease relapse rate and, to a lesser extent, disease progression by targeting peripheral immune cells. However, they largely fail to address Central-Nervous-System-(CNS)-intrinsic pathological processes – especially glial dysfunction – thus leaving a critical gap relevant to disease progression and therapeutic intervention. In this context, neurotrophic factors (NTF) are secreted proteins central for development and maintenance of the CNS. They promote anti-inflammatory, protective phenotypes in astrocytes and microglia, support remyelination by enhancing oligodendrocyte precursor recruitment, maturation and survival, and exert direct neuroprotective effects. Exploring their role in MS offers a novel perspective on neuroimmune crosstalk and prevention of progressive neurodegeneration. In this article, we summarize relevant findings on NTFs in MS, and give an outlook on opportunities and challenges of using these mediators as next-generation disease-modifying therapies.

## 1 Introduction

Multiple Sclerosis (MS) is a chronic inflammatory, demyelinating autoimmune disease of the central nervous system. The disease manifests with neurologic symptoms in a relapsing-remitting (RRMS) as well as a primary (PPMS) or secondary progressive (SPMS) form. It is characterized by the presence and development of multifocal inflammatory lesions in brain and spinal cord (1). T cells play an important role in the disease as they pass the blood-brain-barrier, recognize Central-Nervous-System-(CNS)-specific autoantigens and cause focal demyelinating lesions with axonal damage and reactive astrogliosis (2) characterized by the activation of astrocytes and microglia as well as deposition of extracellular matrix. Recently, long thought beliefs that neurodegeneration typically happens later in the disease course have been increasingly challenged. Research indicates that neurodegeneration occurs early in the disease (3, 4) and contributes largely to disability progression (5, 6). Indeed, the relevance of CNS-intrinsic glial cells in this process has become evident. Glial cells such as astrocytes can lead to the promotion of inflammation, but also contribute to its alleviation by membrane-bound as well as secreted mediators as outlined in a recent review (7).

Effective therapies targeting peripheral immune cells have been successfully established in recent years. However, their efficacy in the progressive course of the disease is limited (2) as progression seems to be mostly associated with intraparenchymal processes within the CNS (8). These intraparenchymal mechanisms are mostly driven by resident glial cells, e.g. astrocytes, microglia and oligodendrocytes, and their complex crosstalk (9). Glial cells are essential for support and protection of neurons and their axons, intercellular communication in the CNS, facilitation of synapse formation and neurotransmission as well as the detection of inflammation and tissue damage (10).

While the proinflammatory and neurotoxic properties of glial cells in MS have been of interest for a long time, only recently has their protective potential become a topic of interest (7, 11). Microglia play an important role in the phagocytosis of myelin debris following demyelination and promote recovery and remyelination by secretion of anti-inflammatory and immunomodulatory cytokines as well as neurotrophic factors (12, 13). In MS, they have been shown to play beneficial roles by formation of a physical barrier at the lesion site as a result of astrogliosis and providing trophic support for neurons and oligodendrocytes by secretion of neurotrophic factors, neuropoietic cytokines and growth factors (7).

Thus, glial cells exert part of their protective functions by the release of so-called neurotrophic factors (7). For reasons of clarity, conciseness and volume, this review focusses on an overview of glial neurotrophic factors, which might be of relevance for future therapeutic and translational strategies. But what exactly are neurotrophic factors?

### 1.1 Neurotrophic factors

Neurotrophic factors (NTFs) comprise a family of secreted proteins which are essential for the development, survival and function of neurons in the nervous system (14). In the prenatal brain, the survival of neurons depends on access to NTFs, while in the adult CNS, they provide a balance between degeneration and regeneration (15). NTFs can be broadly classified into the four neurotrophins, which are Brain-derived neurotrophic factor (BDNF), Nerve growth factor (NGF), neurotrophin 3 and neurotrophin 4, the Glia derived neurotrophic factor (GDNF)-family, members of the Ciliary neurotrophic factor (CNTF)-family as well as the more recently discovered factors Mesencephalic Astrocyte-Derived Neurotrophic Factor (MANF) and Cerebral Dopamine Neurotrophic Factor (CDNF). Several members of growth factor families, e.g. the epidermal growth factor family (EGF), have also been shown to support neuronal survival, but there is no consensus whether these factors fulfill the criteria of classical neurotrophic factors (16).

Neurotrophins are initially synthesized in the endoplasmic reticulum as precursors called proneurotrophins which are then processed into mature neurotrophins (17). The family of neurotrophins exert their effects by binding with high affinity to tropomyosin receptor kinases (Trk, namely TrkA, TrkB and TrkC) as well as the low-affinity p75 neurotrophin receptor (p75NTR). Trk-receptors are transmembrane proteins, consisting of an extracellular domain, a transmembrane region and an intracellular region, which contains the tyrosine kinase (18). Each of the four neurotrophins has a binding specificity for a distinct Trk-receptor. NGF binds to TrkA, BDNF and neurotrophin 4 to TrkB while neurotrophin 4 can bind to all Trk but has the highest affinity for TrkC (18). Trk are widely expressed in neurons, however they are also expressed in glial cells, especially TrkB (19–22) Most other neurotrophic factors also bind to and activate receptor tyrosin kinases (14). In the CNS, neurotrophic factors are secreted by neurons as well as glial cells (23, 24). Neurotrophic factors are implicated in a number of diseases of the CNS, which involve inflammation and neurodegeneration like Parkinson’s disease, Alzheimer’s disease, Huntington’s disease and neuropsychiatric disorders (25–28).

As outlined, NTFs play an important role in modifying inflammation in the CNS and controlling neurodegeneration, which both are central in MS. Therefore, investigating their role in the disease offers a deeper understanding on the pathophysiology of MS, especially in the progressive phase and their neuroprotective and myelinating properties may inspire the development of novel disease modifying drugs. Therefore, in this review, we will outline the current knowledge on the most relevant NTFs in MS. To allow for a more condensed, in depth analysis, we will focus on four classical neurotrophic factors – the neurotrophins BNDF and NGF, GDNF, CNTF as well as the growth factor Hb-EGF with an emphasis on recent data. While Hb-EGF is not a classical neurotrophic factor, it exerts a number of neurotrophic-like functions like tissue regeneration and promotion of neuronal survival and has been studied in the context of inflammatory brain disease (29, 30). Other factors like Neurotrophin-3/-4, Neurturin might also be of interest in MS in the future, their role has been less extensively studied and their functional relevance in MS remains less well designed.

#### 1.1.1 BDNF

**Figure 1:**
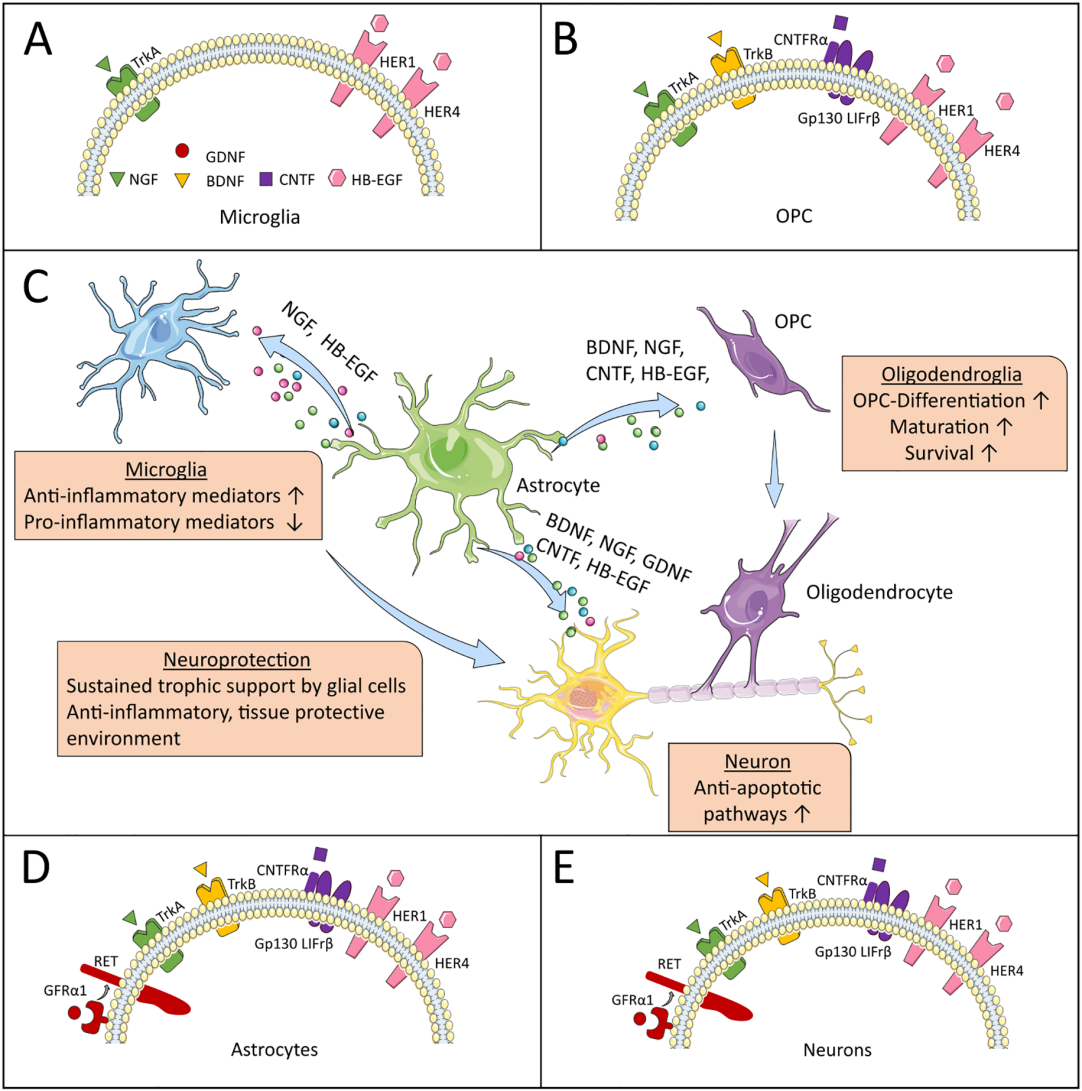
Neurotrophic factors and their receptors. **(C)** highlights astrocytes as central producers of NTFs, illustrating their beneficial effects on specific CNS-resident cells in MS. **(A, B, D, E)** depict the expression patterns of NTF receptors on these target cells, through which the NTFs mediate their supportive functions. The artwork used in this figure was adapted from Servier Medical Art (http://https://smart.servier.com/). Servier Medical Art by Servier is licensed under a Creative Commons Attribution 3.0 Unported License.

Brain-derived neurotrophic factor (BDNF) is one of the most common neurotrophins in the CNS and plays important roles for synaptic development and plasticity, neuronal differentiation, -transmission and -protection (31). In the CNS, it is expressed by neurons, astrocytes, oligodendrocytes and microglia (32–35). Glial expression in the healthy CNS is low (36), however, astrocytes significantly upregulate its expression during inflammatory conditions (7, 37). BDNF is first synthesized in the endoplasmic reticulum as preproBDNF and then cleaved into proBDNF in the Golgi apparatus. BDNF binds its specific receptor TrkB with high affinity, while BDNF, its precursor proBDNF as well as the other neurotrophins of the neurotrophin family bind to p75NTR (38) (**Figure 1A**). Engagement of BDNF with TrkB mainly regulates neuronal survival, differentiation and plasticity, while p75NTR can act pro-apoptotic but can also promote cell survival (39). TrkB is widely expressed in the central nervous system, mainly in neurons, astrocytes and oligodendroglia (36, 40). BDNF signaling regulates the cross-talk between astrocytes and microglia, thus influencing inflammatory mechanisms and exerting anti-inflammatory effects (41). BDNF-activity is altered in some patients with a distinct single-nucleotide polymorphism, where valine is exchanged with methionine at codon 66 in the BDNF gene pro-domain encoding region (Val66Met-polymorphism). This polymorphism has been studied in several disorders, including MS, however, data remains inconclusive as to its disease-exacerbating or protective effects in MS (42, 43). A recent study found no difference in clinical characteristics between the Val66Met polymorphism in MS (44). However, at disease onset, Val66Met-carriers showed reduced cortical thickness (44). Still, the influence of the polymorphism on MRI structural measures remains controversial, as other studies reported beneficial effects on gray matter volume in MS (45–47). Evidence points to epigenetic mechanisms, namely the level of methylation of the BDNF gene playing a more important role than the prevalence of the Val66Met-polymorphism itself. Genetic analysis of 209 MS patients revealed no influence of the polymorphism on the disease course, however, hypomethylation of the BDNF gene in the exonic CpG-site affected by the polymorphism was associated with a higher burden of disability. This indicates an upregulation of BDNF expression as a response to higher inflammatory/disease activity (48).

Indeed, data on BDNF levels in MS-patients showed conflicting results, as some authors described increased, decreased and some unchanged levels in the disease (49–57). A recent meta-analysis, which included thirteen studies with 689 MS-patients, concluded that circulating levels of BDNF are decreased in MS with disease duration correlating negatively with BDNF-levels (58). In clinical relapse, most studies reported an increase of BDNF-concentration (43, 52, 59). However, most of the data is over ten years old and relies on BDNF-levels in peripheral blood mononuclear cells (PBMC) or serum, as BDNF-levels in Cerebrospinal fluid (CSF) are low and ultra-sensitive immunoassays are necessary for its reliable detection (60). A more recent study found a positive correlation between serum BDNF-concentration at baseline and improvement in MS-disability-score EDSS 12 months after relapse (61). In a study with 28 patients with RRMS and 28 healthy controls, serum levels of BDNF negatively correlated with the number of T2-hyperintense lesions in MRI. However, no association was found between T1-positive lesions and Gadolinium-enhancing lesions. Comini-Frota, Rodrigues (62) - the authors of the study - speculate that BDNF controls acute repair processes and is therefore associated with acute demyelination as indicated by T2-lesions. T1-lesions, which mostly account for chronic lesions with chronic neuronal loss, can therefore not be influenced by BDNF. However, this finding cannot explain the missing association with Gad+-lesions in the data, which represent acute demyelination.

BDNF expressing cells are more numerous in actively demyelinating areas and TrkB is upregulated near MS plaques as was shown in a human post-mortem study (63). Preclinical, *in vivo* studies demonstrate that in the absence of BDNF, mice experienced a stronger disease course in experimental autoimmune encephalomyelitis, EAE, the animal model of MS (64, 65). This effect was more pronounced when BDNF was deleted early in the disease (65). This suggests that neurodegeneration already occurs early in the disease and could be ameliorated by neuroprotective agents like BDNF.

BDNF has been shown to play an important role in remyelination. Astrocytes promote oligodendrocyte progenitor cell (OPC) maturation and oligodendrogenesis by secretion of BDNF after white matter damage (66). Knock-down of TrkB in mice reduced myelination/myelin thickness and decreased the proliferative potential of OPCs, highlighting the potential for BDNF in remyelination (21). In accordance with this study, a BDNF-mimetic induced oligodendrocyte differentiation and myelin repair via TrkB in the cuprizone-induced model of toxin-induced demyelination (67). In another study, astrocytes were shown as key players in BDNF-dependent remyelination using the cuprizone model (37). The injection of 2-chloro-5-hydroxyphenylglycine (CHPG), an agonist of the astrocytic metabotropic glutamate receptor 5 enhanced myelination via astrocytic release of BDNF after cuprizone-induced injury in mice (68). As BDNF can hardly pass the Blood Brain Barrier (BBB), Kopec, Kiptoo (69) combined intravenous BDNF-injections with application of the BBB-modulator ADTC5. The combination reduced clinical disease progression and induced oligodendroglia-activation and remyelination in EAE mice (69).

Several immunotherapies have been shown to enhance BDNF levels. Glatiramer acetate, a synthetic mixture of 4 amino acids intended to mimic myelin basic protein, one of the major myelin autoantigens in MS (70), increases BDNF levels (71–73) as well as Interferon-beta (55, 74), even though these agents do not readily cross the blood brain barrier, indicating indirect effects founding these observations. Yet, Fingolimod, which can cross the blood-brain barrier after phosphorylation, has been shown to increase BDNF-levels in patients with MS (75, 76).

In summary, the current data suggests that BDNF controls remyelination after autoimmune inflammatory attacks and is an important factor in glial-derived neuroprotection. Therefore, a better understanding of its role in MS might offer new therapeutic avenues particularly for progressive disease stages characterized by compartmentalized inflammation.

#### 1.1.2 NGF

Nerve growth factor (NGF) was the first neurotrophin discovered in 1950 (77). Its mature form is cleaved from its precursor proNGF (78). NGF mediates its biological function through two receptors, the high-affinity receptor TrkA and the low-affinity p75-receptor (79) (**Figure 1A**). While TrkA mainly activates growth and survival pathways and affects synaptic plasticity modulation, the effects of p75NTR are context specific and the receptor can form complexes with various other receptors, which mediate a great number of different effects, including pro-apoptotic pathways (80, 81). In the CNS, NGF is expressed in neurons, microglia, oligodendrocytes and astrocytes (81–83). In the inflamed brain, NGF-overexpression is induced in neurons and glia cells (84). In EAE NGF, TrkA and p75NTR are upregulated in inflammatory lesions of the spinal cord (85).

NGF mediates protective functions for oligodendroglia, ultimately protecting from demyelination. NGF can act directly on oligodendrocytes, which have been shown to express NGF-receptors TrkA and p75NTR (86, 87). Through activation of the Erk1/2-MAPK-pathway, NGF preserves myelin thickness and leads to its maintenance (88, 89). The synthetic micro-neurotrophin BNN27, which binds specifically to TrkA and p75NTR, protects mature oligodendrocytes against cell death in a cuprizone-induced model of demyelination in a TrkA-dependent manner *in vitro* and protects against myelin loss and reduces microgliosis and astrocytosis in an *in vivo* mouse model. (86). Another recent study, using mixed neural stem cell-derived OPC/astrocyte cultures showed that NGF induces OPC differentiation, maturation and protects them against cell death in the context of oxygen and glucose deprivation. In this mixed culture, astrocytes were the main producers of NGF (87). In microglia, NGF induces a neuroprotective phenotype (90). In accordance with these results, NGF and its receptors co-localize with anti-inflammatory microglia, but not pro-inflammatory microglia phenotypes in the EAE model (85). NGF also induces neuroprotection in astrocytes, as reduced levels of NGF induce a neurotoxic phenotype (91). In a recent study, artificial microvesicles carrying NGF showed beneficial effects in EAE by reducing neurogliosis and stimulating axon regeneration (92).

Taken together, NGF induces protective mechanisms in glial cells and can help protect against demyelination by protecting oligodendroglia.

#### 1.1.3 GDNF

Glia derived neurotrophic factor (GDNF) is a member of the GDNF-family consisting of GDNF, neurturin, artemin and persepin. All four members are part of the TGF-ß superfamily. They exert their effects by activating the transmembrane receptor tyrosine kinase “rearranged during transfection” (RET), which regulates cell survival, differentiation, proliferation, migration, chemotaxis, branching, neurite outgrowth and synaptic plasticity (93) (**Figures 1D, E**). In the “healthy” brain, GDNF secretion is usually low and with neurons as its main cellular source. However, under autoimmune inflammation, its expression is upregulated in glial cells, e.g. astrocytes, microglia and infiltrating macrophages (94–96). So far, studies have shown beneficial effects of GDNF on dopaminergic neurons in Parkinson’s disease (97), striatal projection neurons and cortical neurons in Huntington’s disease (98) and motor neurons, which degenerate in amyotrophic lateral sclerosis (99, 100).

Recent data on GDNF in MS is scarce. In a current study, Sokolowski, Kucharska-Lusina (101) showed reduced gene expression and protein concentration of GDNF in the plasma of MS-patients, indicating impaired neuroprotection. Exposure of astrocytes to the CSF of MS-patients resulted in activation of astrocytes and increased expression of neurotrophic factors GDNF, BDNF and VEGF (102). Interestingly, Jin, Zhang (103) revealed the novel role of SARM1/GDNF-signaling in EAE. SARM1, short for Sterile Alpha and TIR Motif Containing 1 is a member of the Toll/interleukin 1 receptor junction family, is highly expressed in the CNS and is involved in mediating neuronal cell death and axonal degeneration (103). Astrocytic SARM1 promotes neuroinflammation and axonal demyelination in EAE by inhibiting the expression of GDNF and knock-out of SARM1 delayed disease onset and reduced inflammation, demyelination and neurodegeneration by up-regulation of GDNF (103).

Thus, GDNF serves as a neuroprotective agent in the CNS and might offer therapeutic potential to protect against neurodegeneration. However, further studies on the role of GDNF are needed to provide a broader understanding of its roles in MS.

#### 1.1.4 CNTF

Ciliary neurotrophic factor (CNTF) forms part of the IL-6 cytokine family (104). In the CNS, it is primarily expressed by astrocytes, but not by neurons (105, 106). It provides protection for several types of neurons (sensory, sympathetic, motor neurons) and glial cells and plays an important role in brain development and neuronal differentiation (107–109). CNTF has been shown to induce the maturation of astrocytes and oligodendrocytes (7, 110, 111). Its receptor is composed of three components, the non-signaling CNTFRα, which is specific for CNTF, gp130 and LIFrβ, that are shared with other members of the IL-6 family including LIF and IL-6 (112). Binding of CNTF to CNTFRα induces the ß-receptors gp130 and LIFrβ, which then activate signaling cascades including JAK/STAT, Ras/MAP kinase and the phosphatidylinositol 3-kinase pathway (113) (**Figures 1B, D, E**).

CNTF has long been a protein of interest in motoneuron disease due to its protective effect on motoneurons (114). Recent studies investigating CNTF in MS are scarce. In an older study, CNTF receptor expression was enhanced in post-mortem cortical neurons of MS patients and CNTF expression was increased in neurons and astrocytes (108). CNTF has been shown to be expressed in a biphasic manner by astrocytes in a cuprizone model of demyelination, during early demyelination and then again in remyelination (115). It provides protection against demyelination in CNS-disease with more severe EAE-phenotype in CNTF-deficient mice with a strong decrease in OPC count and increase in oligodendrocyte death (116). Intraventricular injection of CNTF in EAE mice reduced expression of inflammatory cytokines TNF-α and interferon-γ and decreased demyelination and neurodegeneration (117). Overexpression of CNTF in Mesenchymal Stem Cells (MSC) and subsequent intravenous injection in EAE mice reduced demyelination, lowered levels of pro-inflammatory cytokines and ameliorated clinical disease course. However, MSC-CNTF were only found in the spinal cord, but not in the brain (118).

In summary, some studies showed a beneficial role of CNTF in MS disease pathology, however, more detailed research of recent time is mostly lacking.

#### 1.1.5 HB-EGF

Heparin-binding epidermal growth factor-like growth factor (HB-EGF) is a member of the epidermal growth factor (EGF) family, which also includes EGF and TGF-α. Its early form is a transmembrane protein called pro-HB-EGF, which is then cleaved by a number of proteases (e.g. ADAMs, MMPs), generating soluble HB-EGF (29). It was first isolated in macrophage-like cells in the early 1990s (119). HB-EGF is a potent stimulator of cell proliferation and migration and targets a number of cells, including peripheral cells including epithelia cells, smooth muscle cells and fibroblasts (120). HB-EGF binds to epidermal growth factor receptors HER1 and HER4, which activate a tyrosine kinase triggering a series of signaling cascades including MAPK and AKT pathways (29) (**Figure 1A**). It is involved in development, homeostasis and tissue growth but also modulates inflammatory functions (121).

HB-EGF is produced by astrocytes during acute stages of neuroinflammation and decreases in later stages of the disease in EAE and in the CSF of patients with RRMS (30, 122). Its expression is induced by hypoxia, whereafter it exerts neuroprotective functions and stimulates neurogenesis (30, 123). Activation of the S1P-receptor, for example by S1P-receptor modulator Fingolimod, induces upregulation of astrocyte-derived Hb-EGF and - among other factors like LIF - protects cultured neurons against excitotoxic cell death (124, 125). In a cuprizone-induced model of demyelination of mice of different ages, intracisternal co-injection of EGF and Hb-EGF increased the proliferation of neural progenitor cells (NPC) in the ventricular-subventricular zone of the CNS, which increased the number of oligodendrogenic NPCs available for remyelination. The production of NPC-derived oligodendrocytes was increased in the corpus callosum following the injection, indicating an important role of HB-EGF in myelination. However, the total density of oligodendrocytes and myelin abundancy remained stable in this study, indicating an equilibrium with other oligodendrocyte sources and a consecutive downregulation of these other sources (126). Linnerbauer, Lößlein (30) could limit neuroinflammation by intranasal delivery of HB-EGF in the EAE model. HB-EGF reduced neuroinflammation through effects on multiple CNS-resident cell types like microglia, protected neurons against the pro-apoptotic effects of TNFα and was shown to support OPC survival and myelination (30). On the other hand, inflammatory conditions induced epigenetic changes in astrocytes by HB-EGF promoter hypermethylation. This suppressed HB-EGF expression and therefore hinders tissue-protective programs, offering a possible explanation for the progressive decrease of HB-EGF in advanced disease stages of MS and EAE (30). Of note, reduced HB-EGF expression in an inflammatory environment could be reversed by treatment with 5-Aza(-cytidine), a clinically approved chemotherapeutic agent and DNA methyltransferase inhibitor, confirming EAE-ameliorating effects of the drug in earlier studies (30, 127, 128).

Thus, HB-EGF might represent a promising factor relevant to disease progression and might serve as a novel therapeutic factor worthy of further study, either as a downstream target by selective demethylation strategies or as a drug itself.

## 2 Translational potential of neurotrophic factors

MS, especially in the progressive disease phase, poses therapeutic challenge as current disease modifying therapies fail to address the complex crosstalk between glial cells, neurons and CNS-infiltrating cells as well as their highly dynamic functions (2, 9).

Highlighting the functional dynamics of astrocytes, depletion of reactive astrocytes in early neuroinflammation increased disease severity in EAE, while depletion of reactive astrocytes during the chronic disease phase ameliorated disease pathogenesis (129, 130). Moreover, astrocytes represent a diverse population of cells with strong spatial and environment-dependent signatures (131). Similarly, microglia are well known for their proinflammatory and neurotoxic roles in neuroinflammation, however, they as well exert a number of protective effects, e.g. by phagocytosis of myelin debris, maintaining myelin health, limiting neurodegeneration, secretion of growth factors and elimination of destructive T-cells (13, 132–134). Considering the diversity of glial responses in MS, specific therapeutic interventions, which modulate distinct glial subsets are needed, since an overly broad approach as well as drug-application at an improper time-point might worsen clinical outcome by also affecting protective glial functions (2). Yet, neurotrophic factors are key players in this neuroimmune crosstalk (7). In the paragraphs above, we have outlined their beneficial effects on inflammation and neurodegeneration in MS (**Table 1**, **Figure 1C**). Their ability to activate anti-inflammatory pathways and to modulate glial cells into a neuroprotective and anti-inflammatory phenotype (90, 91) therefore makes neurotrophic factors interesting candidates for the development of new therapeutic approaches.

**Table 1:** Potential effects of Neurotrophic factors in MS.

Mediator	Cellular source	Cellular targets	Potential effects in MS	References
BDNF	Neurons, Astrocytes, Oligodendroglia, Microglia	Neurons, Astrocytes Oligodendroglia,	Decreased serum levels during clinically stable phases	(49–58)
			Neuroprotective effects and upregulation during relapse	(52, 63–65)
			Supports remyelination and differentiation of oligodendroglia	(21, 66–68)
			Enhanced BDNF-levels by Glatiramer acetate, Interferon-beta and Fingolimod	(55, 71–76)
NGF	Neurons, Astrocytes, Oligodendroglia, Microglia	Neurons, Astrocytes, Oligodendroglia, Microglia	Mediates protective functions for oligodendroglia/protects against demyelination and promotes myelin thickness and maintenance	(86, 87)
			Induction of neuroprotective functions in microglia and astrocytes	(85, 90, 91)
GDNF	Neurons, Astrocytes, Microglia, Infiltrating Macrophages	Neurons (Dopaminergic neurons, Striatal projection neurons, Motoneurons, cortical neurons), Astrocytes	Reduced plasma concentration in MS-patients	(101)
			Delayed disease-onset and reduced neuroinflammation and neurodegeneration in EAE	(103)
CNTF	Astrocytes	Neurons (Motoneurons, cortical neurons), Astrocytes, Oligodendroglia	Protection against demyelination	(116–118)
			Increased CNTF-expression in neurons and astrocytes of MS patients	(108)
HB-EGF	Astrocytes	Neural Progenitor cells, Astrocytes, Oligodendroglia, Microglia	Limits neuroinflammation and protects neurons against pro-apoptotic mediators	(30)
			Supports OPC maturation/proliferation, promotes myelination	(30, 126)
			Enhanced HB-EGF-levels by Fingolimod/S1P-receptor-induction	(124, 125)

**Figure 2:**
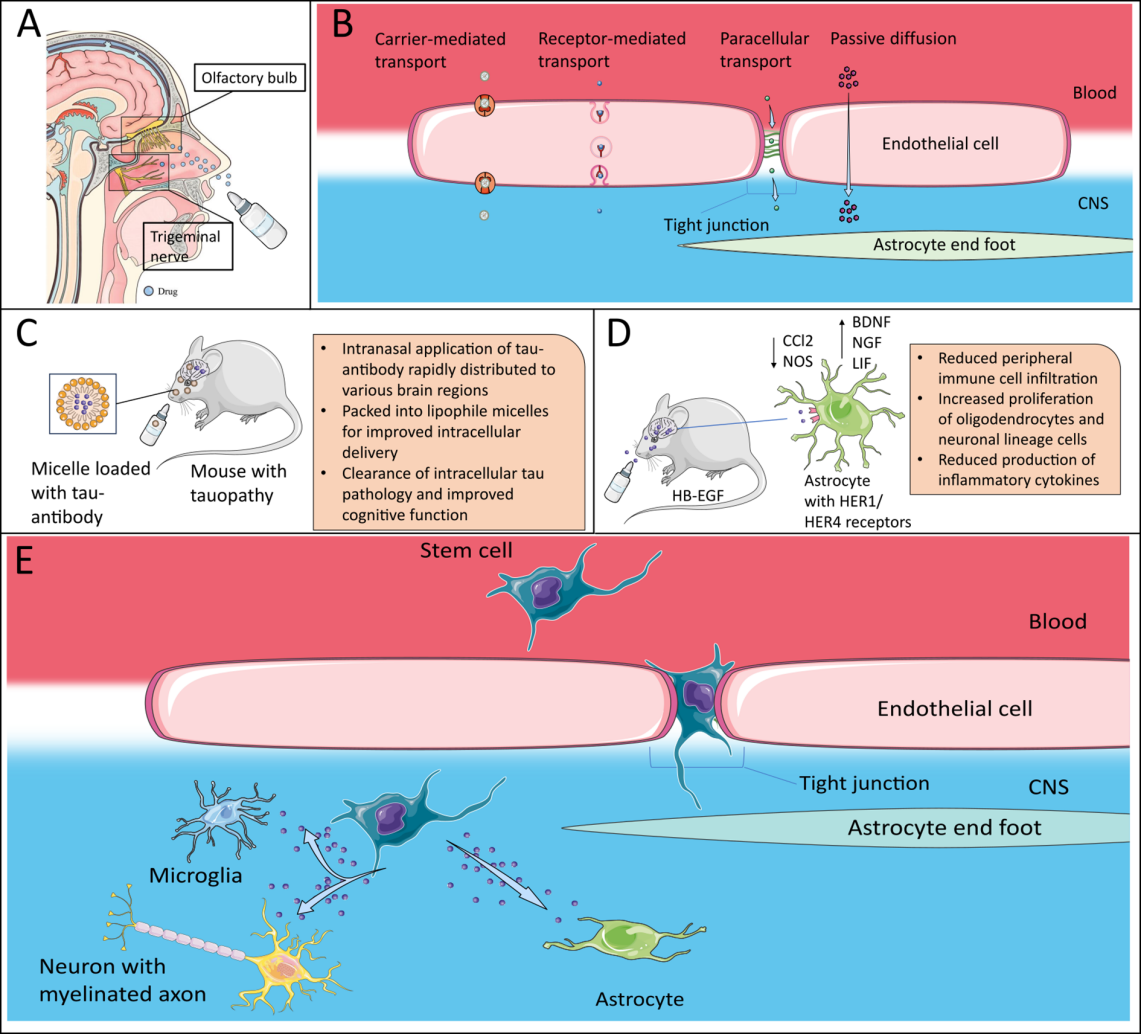
Therapeutic potential of neurotrophic factors. **(A)** Intranasal delivery of drugs via the olfactory nerve system or the trigeminal nerve to bypass the BBB. Efficacy can be further improved by use of nanoparticle formulations. Adapted after (143), which is licensed under CC BY 4.0. **(B)** Nanoparticles can pass the BBB via different mechanisms, which include carrier-mediated transport, receptor-mediated transcytosis and paracellular transport across tight junctions as well as passive diffusion due to their small size. **(C)** Packed into lipophile micelles, intranasal application of an tau-antibody was able to clear intracellular tau pathology (144). **(D)** Intranasal delivery of HB-EGF in mouse model successfully ameliorated EAE-disease course by inducing an anti-inflammatory phenotype in astrocytes, inducing trophic effects in CNS-intrinsic cells and reducing peripheral immune cell infiltration (30) **(E)** Stem cells, e.g. mesenchymal stem cells can be modified to overexpress NTFs. They can cross the BBB, especially in the inflamed brain and release their cargo. The artwork used in this figure was adapted from Servier Medical Art (http://https://smart.servier.com/). Servier Medical Art by Servier is licensed under a Creative Commons Attribution 3.0 Unported License.

Yet, several challenges, however, must be addressed before their clinical application. Indeed, most data on neurotrophic factors stems from *in vitro* or animal models, underlining the need for human-derived data. Diagnosing NTF dynamics in MS patients is complex. Taking BDNF as an example: although it is stored in blood and platelets and therefore measurable in serum, its diagnostic utility for CNS-intrinsic mechanisms is limited (57). In contrast to Neurofilament Light Chain (NFL), a promising biomarker for neurodegeneration increasingly used in MS whose serum levels correlate well with CSF levels, it is unclear whether BDNF levels in serum reflect those in CSF (57). As a result, serum BDNF provides only limited insight into CNS-intrinsic mechanisms. Moreover, NTFs like BDNF and NGF are typically present in the CSF at very low concentrations, often below the detection limits of conventional ELISAs (60, 135). More sensitive assays are necessary for obtaining reproducible and biologically meaningful results, such as Single Molecule Arrays (SIMOA) (60). In clinical practice, CSF sampling via lumbar puncture is generally conducted only during the initial diagnostic work-up when most patients show a relapsing remitting form of the disease. Over time, as more patients transition to SPMS, CNS-intrinsic mechanisms primarily driven by resident glial cells become dominant (9). In a rare case of a MS-patient with available longitudinal CSF-samples Linnerbauer, Lößlein (30) found a continuous decline of HB-EGF levels over the disease course whereas initial HB-EGF CSF-levels in RRMS-patients were increased. This highlights a key limitation: most available CSF-samples are collected during early disease stages, potentially missing critical NTF alterations relevant to the progressive phase of MS. Repeated spinal taps for scientific purposes pose a challenge considering the invasive nature of the procedure (136, 137). Even when obtained, CSF-concentrations reflect global changes across the CNS and cannot resolve cell type-specific or region-specific dynamics in NTF expression. This is a significant limitation, especially given that MS is characterized by focal inflammatory lesions. To address this, human brain tissue samples offer superior spatial and cellular resolution. However, access to high-quality human brain tissue remains limited (138). The increasing establishment of brain banks – which systematically collect and store post-mortem brain tissue – provides valuable opportunities for studying MS-specific mechanisms in human CNS (139). Nonetheless, the success of such efforts depends critically on the quality of sample preservation, particularly for protein and gene expression analyses (139). Therapeutic interventions that target CNS-resident cells have been limited by the challenge of delivering drugs across the BBB, as NTFs pass the BBB only to a limited extent (140, 141). New application strategies might solve this problem. An interesting approach to passing the BBB is intranasal drug delivery, which involves transport via the trigeminal and olfactory nerve systems as direct connectors between the nasal cavity and the CNS (142) (**Figure 2A**). Relevant mechanisms include passive diffusion, paracellular transport, carrier transport and receptor mediated transcytosis (141, 145) (**Figure 2B**). Indeed, neurotrophic factors applied nasally pass the BBB and reach relevant concentrations in the CNS by this method of application (141),. Linnerbauer, Lößlein (30) could limit neuroinflammation in EAE-mice by intranasal delivery of HB-EGF (**Figure 2D**). There are additional examples of successful modification of glial - and specifically astrocyte - dysregulation in EAE via intranasal drug administration. Interferon-ß does not cross the BBB when administered peripherally and exerts its neuromodulatory effects mainly on T-cells and monocytes (146). Intranasal application however, ameliorated EAE disease scores by limiting CNS inflammation by acting on the aryl hydrocarbon receptor (AhR) on astrocytes (147). Similarly, intranasal delivery of pleiotrophin, an astrocyte-derived mediator that reduces pro-inflammatory signaling in astrocytes and microglia and exerts neuroprotective effects reduced disease severity in late EAE (148). Highlighting the potential of this approach, several recent translational and clinical studies beyond the field of MS used this method of delivery and were able to clear intracellular tau pathology in tauopathy mice by intranasal application of an anti-tau-antibody (144) or reduce apathy in frontotemporal dementia patients by intranasal application of oxytocin (149) (**Figure 2C**). Intranasal delivery can further be improved by combination with nanoparticle formulations like nanoemulsions, lipids or polymer particles. This was done in the aforementioned study of an intranasal anti-tau-antibody, which was loaded in micelles (144, 150). Another possible approach might be the use of synthetic nanoparticles specifically targeting Trk-receptor-dependent pathways in the CNS. Drug-carrying nanoparticles can significantly improve the delivery of drugs through the BBB and their biodistribution in the CNS (151, 152). Moreover, the modification of cells to express neurotrophic factors is another possible method of drug-delivery into the brain (153). Stem cells possess a high affinity to sites of injury with active migration from their respective origin. They can cross the BBB, especially during inflammation and have immunomodulatory properties (154–156) (**Figure 2E**). Their homing to inflamed areas can further be improved by modifying homing ligands, either by DNA-transfection or – which is potentially more scalable and cost-effective – pre-treatment with small molecules (157, 158). Pre-treatment of MSCs with Ro-31-8425, a selective protein kinase C (PKC) inhibitor, increased surface expression of CD11a in MSC and improved ICAM-1-dependent cell adhesion at inflammatory sites in mice (157). As PKC-dependent pathways also play a central role in T-cell activation, systemic administration of MSC loaded with Ro-31–8425 further ameliorated disease course in EAE mice compared to naive MSC or free Ro-31–8425 by suppressing antigen-specific proliferation of CD4+ T-cells (159). This further underscores the synergistic potential of stem-cell based drug delivery systems. Intravenous application of genetically engineered MSC overexpressing BDNF successfully increased BDNF-concentration in the CNS of EAE-mice and reduced disease severity (160) (**Figure 2C**). Finally, viral vector-mediated gene delivery might permanently upregulate the cellular production of certain NTFs (153).

Taken together, the translational potential for clinical applications for NTFs remains exciting, however, while strategies how to pass the BBB are on the horizon, little is known about potential deleterious consequences of NTFs in the complex neuroimmune crosstalk in MS for an early use in clinical studies. Further studies *in vitro*, in bio databases and in animal models will need to dive deeper into the complex glial-glial and glial-neuronal interactions mediated by NTFs in MS.
